# Novel opportunities for NGS-based one health surveillance of foodborne viruses

**DOI:** 10.1186/s42522-020-00015-6

**Published:** 2020-06-22

**Authors:** Marion Desdouits, Miranda de Graaf, Sofia Strubbia, Bas B. Oude Munnink, Annelies Kroneman, Françoise S. Le Guyader, Marion P. G. Koopmans

**Affiliations:** 1 IFREMER, Laboratoire de Microbiologie, LSEM/SG2M, Nantes, France; 2 Viroscience Department, Erasmus Medical Centre, Molewaterplein 40, 3015 GD Rotterdam, The Netherlands; 3 Centre for Infectious Disease Control, National Institute of Public Health and the Environment, Bilthoven, The Netherlands

**Keywords:** Next-generation sequencing, Metagenomics, Foodborne virus, Food virology, Human enteric virus, Norovirus

## Abstract

Foodborne viral infections rank among the top 5 causes of disease, with noroviruses and hepatitis A causing the greatest burden globally. Contamination of foods by infected food handlers or through environmental pollution are the main sources of foodborne illness, with a lesser role for consumption of products from infected animals. Viral partial genomic sequencing has been used for more than two decades to track foodborne outbreaks and whole genome or metagenomics next-generation-sequencing (NGS) are new additions to the toolbox of food microbiology laboratories. We discuss developments in the field of targeted and metagenomic NGS, with an emphasis on application in food virology, the challenges and possible solutions towards future routine application.

## Food and viruses: the key issues

Foodborne viral infections rank among the top 5 causes of disease, as estimated in the recent study published by the World Health Organization [1]. Most common causes are noroviruses and hepatitis A, while in some parts of the world hepatitis E is considered to be an emerging foodborne disease. Contamination of food can occur by three different routes. The first route is contamination by infected food handlers, related to hygienic practices, policies regarding sick leave, and awareness among food workers and their employers about food safety. The second route is contamination during production, which is particularly problematic for food consumed raw or after minimal processing. Main categories here are filter-feeding bivalve molluscan shellfish and fresh products, notable leafy green vegetables and berries. Shellfish may retain viruses from contaminated environments, in the case of noroviruses through specific ligands that are quite similar to the ligands for noroviruses on human tissues [2]. For fresh products, contaminated irrigation / growing water, or water used for spraying insecticides, has been well documented as cause of contamination [3]. The third possible source of foodborne viral infection is the consumption of meat from infected animals. Here, the best documented foodborne illness is zoonotic hepatitis E, caused by swine hepatitis E virus genotypes 3 and 4, linked to consumption of specific preparations of meat and liver [4, 5]. Given the globalization of the food chain and the increasing burden on the environment of human sewage contamination events, which is related to severe weather events causing sewage overflows, the ambition to keep viruses out of the food chain is a considerable challenge [6].

## What is the current state of genomic epidemiology in foodborne viruses?

The use of viral genomes in surveillance has been established since the 90s. Due to the often-low copy numbers in foods, sensitive Polymerase Chain Reaction (PCR)-based assays have been developed that target specific regions in the genome that can be used for typing [7, 8]. Surveillance plays a crucial part in linking outbreaks and surveillance networks for foodborne outbreaks were developed to investigate the genomic epidemiology of such viruses.

For norovirus, besides national level surveillance initiatives, including CaliciNet in the USA, NoroNet has aggregated data contributed by participating groups in Europe, Asia, Oceania, and Africa. Established in 1996, Noronet (previously designated the “foodborne viruses in Europe network”) has collected a minimal set of epidemiological data and typing data for norovirus outbreaks into a jointly owned searchable database that also provides some data analyses directly accessible for the users [9]. Although far from perfect, judicious use of the data provided has led to major insights in the field, such as the global virus diversity, the periodic emergence of globally spreading variants associated with increased outbreaks, detection of international food-borne outbreaks, establishment of the importance of viral evolution in the epidemiology of noroviruses, and raised questions about origins and the potential role of food as a driver of evolution. More recently, for Hepatitis A and E, HAVNet and HEVNet were created as bottom up initiatives. These networks were established in 2010 and 2017 respectively [10, 11]. All these platforms allow for collaborations between researchers and public health experts interested in exploring the use of genomic characterization as an add-on to epidemiological investigations and surveillance. In order to be functional, a key element of these databases is that they provide some structuring of metadata and a harmonized annotation of strain types. The benefits of such surveillance systems above the public repositories for sequence data, like GenBank, the European Nucleotide Archive (ENA) and the DNA Data Bank of Japan (DDJB), is that the typing of the sequences is curated, and metadata, such as potential transmission routes, outbreak location and the size of the outbreak can submitted along with the sequences [7, 10–13]. The above-mentioned surveillance networks are also providing several tools that allow easy visualization of the sequences and the associated metadata.

However, compared to the globally agreed take-up of molecular typing tools into surveillance of bacterial foodborne pathogens, adoption has been far less swift for viral pathogens [14, 15]. One reason is the aforementioned low copy numbers on food which necessitate highly specialized laboratories. A second reason is the high diversity of these viruses and the lack of a universal target, compared to the sequencing of ribosomal RNA (rRNA) genes for bacteria or eukaryotes metabarcoding. A third reason is that (PCR-based) virus testing of food has been difficult to interpret due to the lack of cell culture systems allowing establishment of viability of the pathogens [6]. Establishment of viability is needed to pinpoint food as the source of an infection, as the spread of noroviruses and hepatitis A is not limited to the foodborne route. Cell-culture models allowing in vitro replication of these viruses now exist, but are still limited to some strains, often heavy to implement, and may not be sensitive enough to allow detection of infectious virus in food samples [16, 17].

For norovirus genotype identification, typing of specific parts of the genome has been the standard. For this, a part of the ORF1 polymerase and a part of the ORF2 VP1 capsid protein are targeted. Since there is frequent recombination between these regions of the genome, both are typed individually and a dual nomenclature has been implemented [18]. The importance of sequencing the overlapping region of ORF1 and ORF2 to identify novel recombinant viruses has been illustrated by the fact that many of the emerging norovirus strains that caused global outbreaks are novel recombinant viruses. A few examples are GII.Pe-GII.4 Sydney 2012 now GII.4_Sydney_2012[P31], GII.17[P17], GII.4[P16] and GII.2[P16] [7, 19, 20]. For molecular typing of Hepatitis A virus (HAV), partial sequences of the VP1 or VP1/2a or VP3/VP1 regions are standardly used, while the nomenclature is based on the VP1 region [21]. For hepatitis E virus (HEV) the VP1 region is standardly used for typing, but the nomenclature is based on complete genomes [22].

## Current approaches in food virology

The first viral food-borne outbreaks, identified in the late nineteenth century, were evidenced by epidemiological data as routine laboratory testing was limited to electron microcopy which lacks the sensitivity needed for meaningful food testing [23]. The lack of a routinely applicable culture system for noroviruses and HAV sparked a range of studies exploring the use of indicator organisms, notably bacteriophages, as surrogates, with limited success. Therefore, since the sequencing of the complete genome of the prototype norovirus in 1990, molecular biology methods became mainstay in food virology, providing for the first-time tools applicable in this field [24]. Indeed, considering the low infectious dose of some human enteric viruses, only highly sensitive methods can be used. The publication of an ISO-CEN method which includes criteria to prevent false negative test results (failure of virus recovery from the matrix) or false positive results was an important step forward to identify virus prevalence in some matrices [25].

**Fig. 1:**
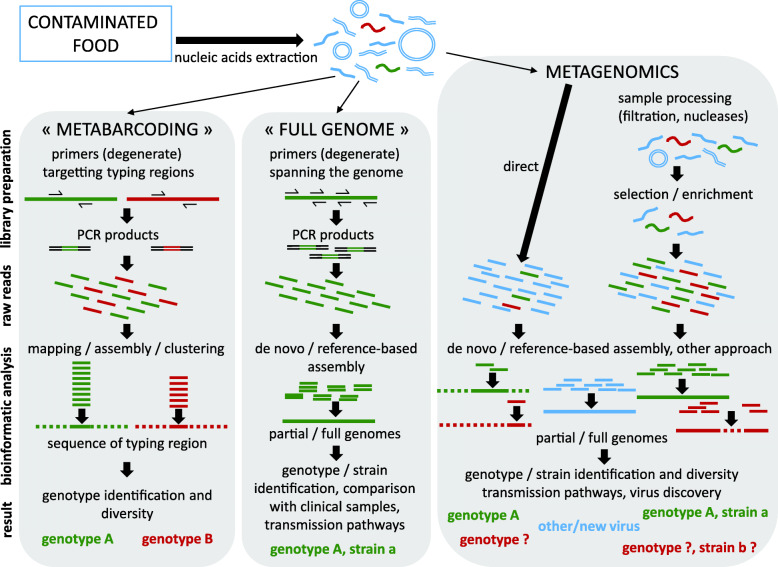
Overview of current NGS strategies for virus sequencing in food. Among nucleic acids extracted from virus-contaminated foods, DNA and RNA material from the matrix and bacteria often prevail (blue), and RNA from the contaminating virus (red, green strains) are scarce. Two strategies use specific primers to focus the sequencing power on a viral contaminant previously identified by other means (qRT-PCR). The “metabarcoding” strategy targets regions of the viral genome commonly sequenced for genotyping. If food products are contaminated by several viral strains belonging to different genotypes (red and green strains), PCR products are synthetized for each strain. Deep sequencing of these amplicons results in a mix of reads corresponding to these different strains. Following bioinformatics analyses (mapping or clustering), each read is assigned to one genotype. This approach allows the identification of strains at the genotype level, as well as an estimation of genotype diversity in the sample, for a given virus. The “full genome” strategy uses several sets of primers to amplify overlapping segments spanning the entire viral genome (around 7–8 kb for most enteric viruses). These PCR products are sequenced together using NGS, generating reads that can be assembled into full or partial viral genomes. Depending on the depth and width of coverage, this can allow the identification of the viral strain, including its genotype classification, and comparison with other samples for analysis of transmission pathways, contamination sources, etc.… A third method, metagenomics, uses random primers for cDNA synthesis. With direct deep sequencing of nucleic acid extracted following fast and simple methods, mostly reads from the matrix or bacterial microbiota (blue) are generated, with often only a limited amount of reads corresponding to the virus (red and green), reflecting the contamination level. Identifying the genotype of the viral strain is possible when such reads fall into the typing region of the virus (green). However, if this not the case virus identification may rely only on sequence comparison with databases (red). Additional steps during library preparation (filtration of bacteria, removal of free nucleic acids, exclusion of the matrix RNA using non-random primers for cDNA synthesis, enrichment in viral sequences using hybridization probes) can result in longer viral sequences (red) and possibly full genomes (green) useful for genotyping and studying the transmission pathways. Importantly, this strategy also allows for the potential discovery of new or unexpected viruses (blue), among the vast diversity of nucleic acid sequenced, depending on the stringency of potential selection/enrichment steps

Next-Generation Sequencing (NGS) or high-throughput deep sequencing refer to successive technologies developed since 2005 that allow for massive, parallel sequencing of DNA fragments. Several studies have now applied NGS for the detection and characterization of the viral diversity in food, using varying strategies. Some target a specific virus or viral families, while others provide a description of the whole RNA or DNA virome (Fig. 1). The first strategy is similar to the metabarcoding approach with deep sequencing of PCR amplicons. Given the wide genetic diversity of viruses, such an approach is limited to closely related viruses or viral families. It was applied to analyze the diversity of norovirus GI and GII genotypes in naturally contaminated oysters from Japan [26], using PCR primers targeting the N-terminal region of the VP1 protein commonly used to determine the norovirus genotype. Combined with microfluidic devices allowing multiplexed PCR, this approach further allowed the detection and genotyping of 11 different human RNA viruses, 4 of them validated on clinical (stool) samples, sewage and laboratory-contaminated oysters [27].

A second strategy also targeting a specific virus is the full-genome sequencing, using deep sequencing of PCR amplicons spanning the entire viral genome. This was applied in clinical samples using primers that target the conserved regions of the 5′- and 3′-end of genomic and subgenomic norovirus RNA [28, 29] and thus span the complete (sub)genomic region, or using multiple sets of primers spanning the complete genome [30]. In food, this strategy was applied on frozen berries linked to HAV-outbreaks in Italy where it allowed the sequencing of a nearly complete HAV IA genome from two samples [31].

Due to the high genetic diversity of the norovirus genus, it is difficult to design primers that are both sensitive and work for a broad range of strains. Therefore, a third strategy is agnostic NGS which uses random primers to circumvents this problem. With these techniques, the list of novel noroviruses has expanded especially for animals [32, 33]. We and others have followed this agnostic metagenomics strategy, sequencing the whole virome of foods linked to foodborne outbreaks [34, 35], samples from the production chain [36–38], or artificially contaminated samples to allow method validation [39–41]. One study also assessed a common, simple workflow for RNA or DNA metagenomics of various matrices (cultured-cell supernatants, stool, tissue and food) for the detection of any kind of pathogen (virus, bacteria and parasites) [42]. In complex matrices, the abundance of reads assigned to human viruses was very low [34, 36, 37, 39–42], or even null for certain viruses [42], reflecting the very low levels of viral contamination in food. Yet, this strategy applied on berries linked to HAV and norovirus outbreaks allowed the sequencing of a nearly-complete HAV IB genome [35] and portions of the norovirus genome closely related to the virus identified in patients [34], respectively. This underlines the potential of NGS to help identify the etiological agents and their possible origin in foodborne outbreaks, but also the limits posed by the detection level of the technique. In naturally-contaminated foods, such as lettuce [37] or meat [36], the authors found few reads assigned to a diversity of mammalian viruses, with some human rotavirus and picobirnavirus in lettuce [37]. The use of viral metagenomics for surveillance and safety-testing of food is thus possible, but to interpret the human health risk posed by the identification of a small number of viral reads remains a challenge. Interestingly, due to the high amounts of sequences from non-mammalian viruses, this approach could also help in the surveillance of circulating antibiotic-resistance genes in phages, and viruses affecting farmed species [36, 38, 43].

## Challenges for implementation of (metagenomic) NGS in food virology

Before NGS can be internationally implemented for detecting foodborne outbreaks, there is a need for standardization and comparison between a plethora of sequencing techniques, as well as methods to analyze these data. For use in clinical settings, the first easy-to-use protocols with precise descriptions of workflows and the use of internal controls such as M2 bacteriophage have been described [44]. Similarly, standardized bioinformatic data processing pipelines have been proposed [45]. However, as NGS is a fast-moving field, with the potential to answer a broad spectrum of questions, there is not always a “one size fits all” protocol. The field should invest in comparison of different techniques and analysis methods to determine both benefits and shortcomings, such as multi institute proficiency testing [46].

### Sample processing

Minimal sample processing is preferred if the type of pathogen is unknown, but it sometimes fails to yield viral sequences due to the overwhelming amount of matrix and bacterial reads [42] (our own observations). The carefully validated and ISO accredited method for sample preparation for virus testing showed some limits when using this in combination with NGS [47]. First, including a virus extraction control in the sample to verify the extraction efficiency is valuable for routine PCR analysis but is not compatible with metagenomic approach, as it may result in an overwhelming abundance of reads corresponding to the control virus at the expense of the target virus. Second, the first step applied to the food matrices to elute the viruses were kept simple to avoid loss of viral particles, but it does not eliminate the matrix background. Other microorganisms and free nucleic acids may be present in the food and eventually might represent the vast majority of the reads obtained after metagenomics. In some studies, evaluating metagenomics for food virology, the authors included sample preparation steps before nucleic acid extraction (Fig. 1), such as filtration or DNase treatment, to minimize the sequencing of the matrix or bacteria [36, 37, 39]. The final yield in human viral reads remained below a few %, but viral pathogens were detected.

### Library preparation and sequencing

Agnostic metagenomics is less sensitive compared to methods that specifically target viruses, and are potentially costlier as it can reduce the number of samples that can be multiplexed. Several techniques have been used to increase the amount of norovirus specific reads to allow for full genome sequencing. By using a poly(A)-capture method to specifically enrich for polyadenylated norovirus RNA, and reduce the amount of non-polyadenylated bacterial RNA before NGS, an approximate 40-fold increase for all tested norovirus genotypes was achieved in clinical samples [48]. However, this approach may not be efficient for food samples with low contamination such as shellfish (our own observations). Alternatively, to enhance virus sequencing, another approach is to use non-ribosomal random primers for cDNA synthesis, i.e. primers selected among random hexamers that do not match rRNA genes from the host or the matrix, like shellfish for example [49, 50]. Targeted enrichment using a custom norovirus whole-genome RNA bait set complementary to and spanning partial or complete reference genomes of 987 norovirus strains also resulted in full genomes from clinical or sewage samples [51–53]. Another strategy to enhance the recovery of human viruses in complex matrices is the capture-based metagenomics (ViroCap), where nucleic acid libraries are enriched in viral sequences using probes targeting all known vertebrate viruses [54]. We successfully applied this technique for the sequencing of norovirus and other human enteric viruses in environmental and shellfish samples [41]. For all of the above, a critical element in the primer/probe design is the choice of a reference virus set that is sufficiently diverse to be able to “catch” all strains from this genetically highly diverse genus. These methods likely have higher detection limits, and therefore may not be feasible for the testing of food with lower levels of contamination.

So far, the ISO accredited method remains compatible with amplicon-based metabarcoding or amplicon based full-genome sequencing, where the target virus is amplified by specific primers (Fig. 1). To enable sensitive amplicon-based sequencing it is likely that genogroup and perhaps even genotype specific primers should be developed. However, when using amplicon-based sequencing novel or divergent norovirus strains might be missed.

### Data analysis

Following high throughput sequencing, several hundred thousand to several million reads are produced for each sample. Processing and analyzing such large amounts of data pose computational demands and requires knowledge of bioinformatics in order to select the most performing and adapted tool while reducing the analysis time. Many tools have been developed and some programs are available online, making the analysis of metagenomic sequences accessible to scientists, like RIEMS [55], JOVIAN (ms in preparation), and Genome Detective [56]. However, the choice of methods is rapidly expanding, depends on the specific applications, the sequencing technology, and is not standardized [57].

Among the different steps in metagenomic sequence analysis, one of the major challenges is the assembly of reads into full genomes or long contigs. Most metagenomics studies conducted on food performed de novo assembly, which allows agnostic treatment of the data without prior knowledge of the viral species to be found, and favors the discovery of new sequences [34, 36, 37, 40–42]. However, the bioinformatics strategy used to analyze sequencing data may greatly affect the results [40]. Yang et al. compared different assembly strategies on the RNA virome data obtained from artificially norovirus/HAV-contaminated celery, and reported better performance of reference based-assembly or in-house k-mer tool using norovirus and HAV references, to identify norovirus and HAV reads that were missed by classical de novo assembly [40].

The abundance of starting data and the variability of viral genomes make the assembly step a real computational challenge. One strategy to reduce computing efforts includes in silico normalization (BBnorm) by filtering excess coverage to eliminate redundant information and to reduce the complexity of the sample before starting de novo assembly [58]. Another approach is a read based annotation with for instance Kaiju, to go beyond the assembly step by translating the short reads from nucleotide sequences directly into amino acids for a direct identification in a database containing microbial and viral protein sequences [59]. Recently, another strategy (Plass) proposes to convert reads into proteins before assembling into longer sequences, reducing variability and facilitating genomic identification [60]. However, it should be noted that these two approaches may lead to a simplification of the output information and potentially be less effective to study genome variants.

For amplicon-based sequencing mostly reference-based alignment or mapping to reference sequences are performed. There are several different tools available for alignment, like minimap2 [61] and kma [62], or mapping, such as BWA [63]. Alternatively, similar to 16S metabarcoding analysis, reads can be clustered into operational taxonomical units (OTU) based on their similarity using FROGS [64] or QIIME pipelines [64, 65]. OTU sequences can then be identified by comparison to a database using Blast or the online virus typing tools.

### Classification and typing of sequences

Classification of foodborne viruses is performed based on short and relatively conserved sequences (polymerase and/or capsid). The rationale for this is that they contain relatively conserved sequences and allow typing of a diverse range of viruses with reasonable sensitivity. This is essential since noroviruses are extremely diverse, but levels of contamination needed to produce infection can be low. For norovirus and other foodborne viruses, the realization is now coming that non-amplicon based NGS of samples with low amounts of contamination will result in fragments that do not necessarily overlap with the selected typing regions. Therefore, it is anticipated that future re-classification of foodborne viruses will be relying on full genome sequences, rather than targeted regions to enable classification of all regions [19].

## How NGS can be useful for food-testing laboratories

Detection of viruses in food is not mandatory but recommended, as the European Food Hygiene package (EU-178/2002) stipulates that ‘dangerous food cannot be marketed’. Since the publication of the ISO/CEN 15216–1 method for virus detection, countries and even private companies have begun to implement virus testing in food. For routine testing an approach such as metabarcoding can be performed on nucleic acid extracts obtained using the ISO method and can be of interest to characterize the contaminant strains. This can be of special interest for example for norovirus as some strains differ in their ability to cause disease [12]. However, full genome or long sequence characterization and/or agnostic approach will be essential either to trace the contaminating event or to identify emerging variants or viruses. In the case of food-related outbreaks, identification of all viral strains present in the sample may help to prevent further disease in humans (for example in a case of a sample with co-contamination of norovirus and hepatitis A virus). Being able to identify the different viruses, and eventually other micro-organisms such as bacteria may help to understand the impact of a combination of pathogens in observed disease. The methods and results described above are mostly based on Illumina sequencing. Nanopore sequencing has not been used as much for foodborne viruses, but it has the potential to constitute an important step for food safety as results can be obtained rapidly and it can be implemented easily in a laboratory at relatively lower cost compared to Illumina sequencing.

## Sharing of pathogen data between laboratories is a precondition for genomic epidemiology of foodborne viruses

Systematic sharing within laboratory networks takes place in pathogen specific, mostly password protected databases such as NoroNet, HAVNet and HEVNet [7, 10, 11]. In these networks, protocols and tools have been developed to ensure harmonization of sequencing and nomenclature. Submitted sequences are not always accompanied by the agreed structured minimum metadata. There can be several possible reasons for this: 1) availability of metadata is often limited, as many laboratories do not receive information beyond time and place of the samples collected. 2) the submission to the data sharing databases is not part of the core business of these labs, and therefore may be done batch-wise with a certain delay. 3) the sharing of metadata, especially across domains (veterinary/food/health) has real or perceived legal barriers [66, 67]. 4) participation is voluntary and not funded, and participants may lack resources to provide data or wait for new funding to provide sequence data.

Even with these limited and incomplete datasets many new insights into the genomic epidemiology have been generated and international outbreaks have been identified. This is why these networks remain active, long after the initiating EU projects, and corresponding financing, have stopped. Such an approach may also be useful for the EURL network to advice National laboratories and will provide data for further investigation and risk analysis or regulation bodies.

With the transition from Sanger to Next generation sequencing, additional challenges surface for data sharing. Besides the consensus genomes or contigs of target pathogens, which are inferred by bioinformatics analysis, also the raw read files themselves are of interest to collect in a shared database. The current pathogen specific databases are not suitable for this large amount of metagenomics data, as it consists of sequences of many viral and non-viral species. The translation of these raw data into useful information for genomic epidemiology requires large computer resources, a new type of metadata and specific bioinformatics expertise. In viromics, standardized methods with validated parameters/cut-offs are not yet available. These challenges are addressed in several international initiatives such as the H2020 funded project COMPARE, where centralized datahubs are developed, raw NGS data can be uploaded, standardized checklists for metadata can be used and data mining tools will be available, which are tested and validated by the consortium, before the sequence data will become publicly available [68]. With initiatives like this, reliable reference databases are built and a storage and analysis infrastructure will become available for everyone.

## Conclusions and future directions

Next generation sequencing has entered the field of food virology, and is widely used experimentally to assess the possible role in outbreak investigations, monitoring of food safety, and source tracking of pathogens. Specific challenges are the low-level contamination that is commonly observed in foodborne viral outbreaks, the complexity and diversity of food matrices, the diverse range of viruses involved, and the lack of a single catch all protocol for virus detection. Nevertheless, promising first results have been obtained that show the potential added value, and we expect to see considerable investment in method development and validation, similar to the early days of PCR-based food testing. Reducing costs and increasing equity in access to novel sequence technologies, analysis workflows, and trusted data sharing platforms should be prioritized to make full advantage of the promising new technologies.

## Data Availability

Not applicable.

## Abbreviations

NGS: Next-generation-sequencing
PCR: Polymerase chain reaction
rRNA: ribosomal RNA
GI: Norovirus genogroup I
GII: Norovirus genogroup II
VP: Viral protein
HAV: Hepatitis A virus
HEV: Hepatitis E virus
DNase: Desoxyribonuclease
OTU: Operational taxonomy unit
EU: European Union
EURL: European Union Reference Laboratory
